# The role of pulmonary arterial hypertension-targeted therapy in systemic sclerosis

**DOI:** 10.12688/f1000research.20313.1

**Published:** 2019-12-19

**Authors:** Michael H Lee, Todd M Bull

**Affiliations:** 1 Division of Pulmonary Sciences and Critical Care Medicine, University of Colorado School of Medicine, Colorado, USA

**Keywords:** Pulmonary arterial hypertension, pulmonary hypertension, systemic sclerosis

## Abstract

Pulmonary arterial hypertension, categorized as group 1 pulmonary hypertension by the World Health Organization classification system, represents a major complication of systemic sclerosis resulting from pulmonary vascular involvement of the disease. The high mortality seen in systemic sclerosis-associated pulmonary arterial hypertension is likely due to the impairment of right ventricular systolic function and the coexistence of other non-group-1 pulmonary hypertension phenotypes that may negatively impact clinical response to pulmonary arterial hypertension-targeted therapy. This review highlights two areas of recent advances regarding the management of systemic sclerosis patients with pulmonary hypertension: the tolerability of pulmonary arterial hypertension-targeted therapy in the presence of mild to moderate interstitial lung disease and the potential clinical significance of the antifibrotic effect of soluble guanylate cyclase stimulators demonstrated in preclinical studies.

## Introduction

Systemic sclerosis (SSc) is a systemic rheumatic disease characterized by autoimmunity, vascular injury, and unchecked collagen synthesis leading to fibrosis of the skin and internal organs ^1, 2^. Based on the extent of skin fibrosis, SSc has been classified as either limited cutaneous or diffuse cutaneous phenotype ^3^, with diffuse cutaneous SSc characterized by earlier and more frequent internal organ involvement ^4^. The term “systemic sclerosis sine scleroderma” is sometimes used to refer to SSc with visceral organ involvement in the absence of cutaneous manifestation ^5, 6^.

One major complication of SSc is pulmonary arterial hypertension (PAH). Historically, PAH has been quoted as one of the leading causes of death in patients with SSc ^7– 11^. PAH represents group 1 pulmonary hypertension (PH) in the World Health Organization (WHO) classification system, characterized by elevated mean pulmonary arterial pressure (mPAP) ≥25 mmHg with elevated pulmonary vascular resistance (PVR) ≥3 Wood units and normal pulmonary capillary wedge pressure (PCWP) ≤15 mmHg in the absence of etiologies known to cause other groups (2–5) of PH ^12^. More recently, mPAP >20 mmHg has been proposed as a new definition of precapillary PH, which includes PAH ^13^. Approximately 10% of SSc patients (under the original definition) are affected by PAH ^10, 14– 16^, and a similar prevalence was re-demonstrated in a recent study ^17^. While SSc-associated PAH (SSc-PAH) comprises a major proportion of PAH registries ^18^, less is known about its incidence, with one study reporting 0.61 cases per 100 patient-years ^19^.

SSc-PAH, which can complicate both limited and diffuse cutaneous subtypes of SSc ^19, 20^, suffers from a particularly poor prognosis. The presence of PAH predicts early death in SSc ^21, 22^, and mortality associated with SSc-PAH is higher than that seen in other PH groups and even other subtypes of PAH ^23^. Multiple studies demonstrated poorer survival in SSc-PAH compared to idiopathic PAH (IPAH) despite less pronounced hemodynamic derangement ^23– 29^. A recent retrospective study of 375 patients showed similar findings (median survival of 3 years in SSc-PAH versus 7.8 years in IPAH), and the difference in mortality was attributed to older age and more pronounced gas exchange impairment in SSc-PAH ^30^. Another plausible explanation for the high mortality in SSc-PAH is reduced contractility of the right ventricle (RV). A number of retrospective studies have consistently documented more impaired RV systolic function in SSc-PAH compared to that in IPAH for a given RV afterload ^31, 32^, and the RV function appears to improve with PAH-targeted therapy in IPAH but not in SSc-PAH ^33^. SSc-PAH is associated with worse outcomes and higher mortality, even when compared to other connective tissue disease-related PAH (CTD-PAH), suggesting that SSc-PAH represents a unique phenotype that perhaps should be managed differently to other subgroups of CTD-PAH ^10, 25, 34^.

The Ambrisentan and Tadalafil in Patients with Pulmonary Arterial Hypertension (AMBITION) trial was a recently published randomized double-blind multicenter study that compared upfront ambrisentan and tadalafil combination therapy to monotherapy in 500 treatment-naïve participants with WHO functional class II–III PAH ^35^. Clinical failure (defined as death, hospitalization for PAH, progression of disease, or unsatisfactory clinical response) was observed in 18% of those receiving the combination treatment compared to 31% of monotherapy recipients (hazard ratio of 0.50). After 24 weeks of intervention, the combination therapy was associated with improvements in secondary outcomes, including the 6-minute walk distance and N-terminal pro-brain natriuretic peptide levels, compared to pooled monotherapy. Other recent studies suggest that PAH-targeted therapy, such as the ambrisentan and tadalafil combination treatment, riociguat, and selexipag, likely confers clinical benefit in the subgroup of CTD-PAH patients ^36– 38^. The optimal medical therapy for the subset of SSc-PAH patients, however, is less defined ^39^, as demonstrated by the SSc-PAH-associated mortality that remains high ^22, 40^ and largely unchanged over time ^29, 34, 41, 42^. For instance, a recent study from the REVEAL registry (the Registry to Evaluate Early and Long-term PAH Disease Management) demonstrated that 5-year mortality in subjects newly diagnosed with SSc-PAH can be as high as 60% ^41^. Despite improved understanding of predictors of mortality in SSc-PAH ^34, 43, 44^ and the development of new prognostic models specifically validated in SSc-PAH cohorts ^45– 47^, clinical outcomes of SSc-PAH remain rather disappointing ^48^.

## Innate heterogeneity of pulmonary hypertension in systemic sclerosis

The poor clinical outcome and suboptimal response to treatment observed in SSc-PAH likely reflect the various phenotypes of PH that can coexist in SSc, precluding straightforward identification of those SSc patients with isolated PAH ( Table 1) ^49^. SSc is often complicated by fibrotic interstitial lung disease (ILD) ^50– 52^, more commonly in diffuse cutaneous SSc than in limited cutaneous SSc ^53^. The fibrotic ILD can be further complicated by neuromuscular weakness and chronic aspiration ^54^, contributing to the development of hypoxemia-induced (WHO group 3) PH. Unselected use of PAH-specific therapy with vasodilatory effect in the presence of ILD can exacerbate ventilation–perfusion (V/Q) mismatch, resulting in aggravation of hypoxemia and secondary PH ^55^.

**Table 1.** Mechanisms of pulmonary hypertension in systemic sclerosis.

World Health Organization group	Mechanism of pulmonary hypertension
1	• Connective tissue disease-associated pulmonary arterial hypertension • Portopulmonary hypertension
1′	• Pulmonary veno-occlusive disease • Pulmonary capillary hemangiomatosis
2	• Myocardial fibrosis • Accelerated atherosclerosis • Kidney failure
3	• Interstitial lung disease (combined pulmonary fibrosis and emphysema)
4	• Venous thromboembolism/chronic thromboembolic pulmonary hypertension (antiphospholipid antibodies)

One form of ILD increasingly recognized in SSc is combined pulmonary fibrosis and emphysema (CPFE) ^56– 58^. Although smoking history places SSc patients at a higher risk of developing CPFE ^59^, it is also observed in never-smokers and those with minimal tobacco history ^57^. A significant portion of SSc patients with CPFE go on to develop PH ^60^, illustrated by a recent study demonstrating a higher incidence of precapillary PH and higher mortality in 36 SSc-CPFE patients compared to 72 SSc-ILD patients without emphysema ^59^. Emphysema in CPFE can cause an additional reduction in carbon monoxide diffusing capacity (DLCO) and lung volume pseudo-normalization, and these physiologic consequences of CPFE can potentially compromise the diagnostic accuracy of PH in SSc patients with fibrotic ILD ^57^.

Under-recognized pulmonary venous hypertension in SSc is also believed to underlie the poor response to treatment and high mortality seen in SSc-PAH. Pathologic studies of lung tissues from SSc-PAH patients demonstrate fibrosis of veins and venules resulting in pulmonary veno-occlusive disease (PVOD)-like changes (WHO group 1) ^61, 62^. In patients with SSc-PAH, the presence of computed tomography findings suggestive of PVOD has been associated with pulmonary edema in response to vasodilator therapy and worse clinical outcomes ^63, 64^. Even more rarely, patients with SSc can suffer from pulmonary capillary hemangiomatosis (PCH), a disease that is now thought to be related to PVOD in its etiology ^65^. Other unrelated mechanisms by which SSc causes post-capillary PH and precipitates pulmonary edema include myocardial fibrosis ^66– 68^ and ischemic heart disease due to accelerated atherosclerosis (WHO group 2) ^69, 70^. Subtle left ventricular diastolic dysfunction due to occult fibrosis of the myocardium may not be apparent and become unmasked with fluid challenge during cardiac catheterization ^71^.

Many studies have also documented the increased risk of venous thromboembolism (VTE) in SSc, which predisposes the patient to developing chronic thromboembolic PH (CTEPH, WHO group 4) ^72– 74^. There is an increased prevalence of antiphospholipid antibodies in SSc, although it remains unclear whether these antibodies represent the autoimmune nature of SSc-PAH, under-recognition of CTEPH, or both ^75– 78^. SSc is also associated with primary biliary cholangitis, which may subsequently produce portopulmonary hypertension ^79, 80^. These various phenotypes of PH often coexist in SSc, and they underscore the difficulty in selecting the right patient population who will benefit from PAH-targeted therapy or even anticoagulation.

## Pulmonary arterial hypertension-targeted therapy in systemic sclerosis patients with concurrent interstitial lung disease and precapillary pulmonary hypertension

The role of PAH-targeted therapy in SSc patients with both ILD and precapillary PH represents a major area of uncertainty and interest to both clinicians and researchers. SSc often involves ILD ^50– 53^, and the presence of concurrent ILD is associated with increased mortality in SSc patients with precapillary PH ^10, 42, 81– 83^. While PAH-specific drugs may alleviate the increased PVR resulting from pulmonary arteriopathy of SSc, the often-coexisting ILD in this patient population has raised the concern of exacerbating V/Q mismatch as more blood flows to the non-ventilated parts of the lungs ^55^. As a result, there has been uncertainty regarding the tolerability of PAH-targeted therapy in SSc-associated precapillary PH owing to the high prevalence of concurrent ILD. Contrary to this historical perspective, however, two recent studies illustrate that PAH-targeted therapy may be well tolerated in the presence of ILD.

As previously mentioned, the AMBITION trial was a randomized double-blind study that demonstrated that the upfront combination therapy with ambrisentan and tadalafil protected treatment-naïve PAH patients from clinical worsening compared to monotherapy ^35^. A recent *post hoc* analysis of the AMBITION trial showed that the combination PAH-targeted therapy conferred similar clinical benefit in the subgroup of SSc-PAH patients ^36^. Of the 118 subjects with SSc-PAH, clinical failure (defined as death, hospitalization for PAH, progression of disease, or unsatisfactory clinical response) was seen in 21% of those receiving the combination treatment compared to 40% of monotherapy recipients (hazard ratio of 0.44 with 56% risk reduction). In the SSc-PAH subgroup, the combination therapy led to significantly improved outcomes compared to what had been previously reported in the general SSc-PAH patient population. While the AMBITION trial specifically targeted subjects with PAH, it is notable that the participants were allowed to have mild to moderate lung disease (total lung capacity [TLC] ≥60% of predicted normal, forced expiratory volume in 1 second [FEV1] ≥55% of predicted normal) ^35^. Therefore, it is probable that a substantial portion of the SSc-PAH subgroup had some degree of ILD or even SSc-ILD-PH, although the presence of severe ILD would have been unlikely given that the average TLC was 90% of predicted ^36^. The subgroup analysis did not investigate the effect of the combination therapy on hypoxemia and gas exchange; however, the study suggests PAH-targeted therapy with vasodilatory effect can be well tolerated in SSc-PAH patients with concurrent non-severe ILD, and it may be unnecessary to exclude a modest degree of ILD prior to trialing PAH-targeted therapy.

A more recent single-center cohort study evaluated response to PAH-targeted therapy in 29 SSc patients who had both right heart catheterization-proven PH and ILD visualized on high-resolution computed tomography of the chest ^84^. The ILDs in these patients were physiologically mild (forced vital capacity [FVC] 70.3% predicted, TLC 84.7% predicted, DLCO 43.1% predicted), followed either nonspecific interstitial pneumonia (NSIP) or usual interstitial pneumonia (UIP) pattern, and affected >20% of the lungs in 65.5% (19 of 29) of cases. The majority of the participants had mild (mPAP 25–35 mmHg) or moderate (mPAP 35–45 mmHg) PH, and six of the 29 had post-capillary PH with PCWP >15 mmHg. A total of 24 of the 29 patients were treated with PAH-targeted therapy, and they tolerated a single, dual, or triple-agent PAH regimen well. Importantly, the PAH-targeted treatment did not worsen V/Q mismatching, again supporting the notion that PAH-specific therapy can be safely used in SSc patients with non-severe ILD. Given the non-randomized nature of this evidence, however, more robust, large-scale, randomized studies are needed before firm conclusions and generalized clinical applications can be made.

## Soluble guanylate cyclase stimulators in systemic sclerosis associated with pulmonary arterial hypertension

Soluble guanylate cyclase (sGC) stimulators, another class of PAH-targeted drugs, have recently gained interest for their potential use in SSc-PAH. In addition to vasodilation, sGC stimulation and a resultant increase in the cyclic guanosine monophosphate level produce an antifibrotic effect by inhibiting non-canonical TGF-β signaling pathways ^85^. In mice, the sGC stimulator BAY 41-2272 prevented and reversed skin fibrosis ^86^. Similarly, riociguat, another sGC stimulator in clinical use, was shown to be effective against skin and gastrointestinal tract fibrosis in multiple mouse models, with its antifibrotic effect more pronounced than that of sildenafil ^87^.

Since SSc is characterized by fibrosis as well as vasculopathy, sGC stimulators with both antifibrotic and vasodilatory effects are considered promising candidates for the treatment of SSc-PAH. Pulmonary Arterial Hypertension Soluble Guanylate Cyclase-Stimulator Trial (PATENT)-1 was a double-blind randomized international study that established the efficacy of riociguat in PAH patients, which was extended to PATENT-2 for the assessment of long-term effects of riociguat ^88, 89^. A recent subgroup analysis of the two trials demonstrated in SSc-PAH patients that riociguat was associated with improvement in cardiac index and reduction in PVR at 12 weeks, and possibly with an increase in the 6-minute walk distance compared to placebo ^37^. Another case series described three patients whose SSc-PAH improved after phosphodiesterase-5 inhibitors were replaced with riociguat ^90^. It remains uncertain, however, whether the antifibrotic effect of riociguat confers additional clinical benefit specific to SSc-PAH.

Despite these seemingly favorable outcomes, the safety and tolerability of riociguat in SSc-PAH patients with concurrent ILD are unclear. In a pilot study of 22 patients with concurrent ILD (TLC >30% of predicted) and treatment-naïve precapillary PH, the use of riociguat was associated with lower PVR and higher cardiac output, suggesting riociguat can safely provide similar hemodynamic benefit in patients with SSc-ILD-PH ^91^. However, in a recently published multicenter randomized trial, Riociguat for Idiopathic Interstitial Pneumonia-Associated Pulmonary Hypertension (RISE-IIP), riociguat was associated with harm and increased mortality in patients with precapillary PH and idiopathic (therefore non-CTD) ILD (mean TLC 66% of predicted) ^92^. While RISE-IIP did not involve SSc or other CTD patients, the demonstrated negative impact of riociguat in patients with significant lung disease will likely deter future attempts to prospectively investigate sGC stimulation in SSc-ILD-PH, barring new, convincing observational data favoring its use in this patient population. Lastly, given that riociguat is effective in CTEPH ^93^ and SSc patients are predisposed to VTE, another interesting question regards the potential role of sGC stimulation in SSc patients affected by CTEPH.

## Conclusion

PAH-targeted therapy appears to be well tolerated and beneficial in SSc-PAH patients with its safety further questioned in the coexistence of ILD. Challenges still remain in choosing the patient population best suited for PAH-specific medications. The distinction between SSc-PAH with ILD (WHO group 1) and SSc-ILD-PH with increased PVR (WHO group 3) is arbitrary and remains undefined. Similarly, the severity of ILD beyond which PAH-targeted therapy becomes harmful is unknown, and this threshold may in part depend on the type of ILD, patient demographics, and other comorbidities. Each SSc patient with PAH or ILD-PH may develop varying degrees of other confounding complications that can independently cause PH (e.g. myocardial fibrosis or PVOD-like changes) either before or even during PAH-targeted treatment, precluding reliable patient selection and accurate response assessment. For example, it is likely prudent to avoid indiscriminate use of PAH-targeted therapy in the presence of significant left-sided heart failure in order to prevent the potential precipitation of pulmonary edema. Furthermore, how rigorously and frequently SSc patients with established PH should be screened for other associated causes of PH is unclear. High-quality, randomized trial-based data are lacking, partially owing to the rarity of the disease.

Novel therapeutic approaches may clarify the unresolved treatment strategies in this patient population. Inhaled PAH-targeted therapy has the theoretical advantage of improving perfusion only in the well-ventilated areas of the lungs, thereby preventing worsened V/Q mismatch that may be seen with systemic administration of the medication. Based on this hypothesis, an ongoing clinical trial (NCT02630316) looks at the role of inhaled treprostinil in ILD-PH patients, the results of which may be applicable to SSc patients with concurrent precapillary PH and ILD. Early evidence suggests the possibility of using gene expression profiling to predict clinical course and monitor response to PAH-targeted therapy in SSc patients, although more data are needed until clinical applicability can be reached ^94– 96^. While we await answers to the aforementioned questions, the decision to implement PAH-targeted therapy in SSc will have to remain individualized, incorporating both the clinician’s experience and the patient’s values.

## Abbreviations

AMBITION, Ambrisentan and Tadalafil in Patients with Pulmonary Arterial Hypertension; CPFE, combined pulmonary fibrosis and emphysema; CTD, connective tissue disease; CTD-PAH, connective tissue disease-related pulmonary arterial hypertension; CTEPH, chronic thromboembolic pulmonary hypertension; DLCO, carbon monoxide diffusing capacity; ILD, interstitial lung disease; IPAH, idiopathic pulmonary arterial hypertension; mPAP, mean pulmonary artery pressure; PAH, pulmonary arterial hypertension; PATENT, Pulmonary Arterial Hypertension Soluble Guanylate Cyclase-Stimulator Trial; PCWP, pulmonary capillary wedge pressure; PH, pulmonary hypertension; PVOD, pulmonary veno-occlusive disease; PVR, pulmonary vascular resistance; RISE-IIP, Riociguat for Idiopathic Interstitial Pneumonia-Associated Pulmonary Hypertension; RV, right ventricle; sGC, soluble guanylate cyclase; SSc, systemic sclerosis; SSc-PAH, systemic sclerosis-associated pulmonary arterial hypertension; TLC, total lung capacity; V/Q, ventilation–perfusion; VTE, venous thromboembolism; WHO, World Health Organization. ****
